# The 1895 Ljubljana earthquake: can the intensity data points discriminate which one of the nearby faults was the causative one?

**DOI:** 10.1007/s10950-018-9743-z

**Published:** 2018-05-30

**Authors:** Lara Tiberi, Giovanni Costa, Petra Jamšek Rupnik, Ina Cecić, Peter Suhadolc

**Affiliations:** 1 Dipartimento di Matematica e Geoscienze, University of Trieste, SeisRaM group, Trieste, Italy; 2 Geological Survey of Slovenia, Ljubljana, Slovenia; 3 Agencija RS za Okolje, Ljubljana, Slovenia

**Keywords:** Macroseismic data, Ground motion scenarios, Ground motion prediction equations, 1895 Ljubljana earthquake, Ground-motion to intensity conversion equations

## Abstract

The earthquake (Mw 6 from the SHEEC defined by the MDPs) that occurred in the central part of Slovenia on 14 April, 1895, affected a broad region, causing deaths, injuries, and destruction. This event was much studied but not fully explained; in particular, its causative source model is still debated. The aim of this work is to contribute to the identification of the seismogenic source of this destructive event, calculating peak ground velocity values through the use of different ground motion prediction equations (GMPEs) and computing a series of ground motion scenarios based on the result of an inversion work proposed by Jukić in 2009 and on various fault models in the surroundings of Ljubljana: Vič, Želimlje, Borovnica, Vodice, Ortnek, Mišjedolski, and Dobrepolje faults. The synthetic seismograms, at the basis of our computations, are calculated using the multi-modal summation technique and a kinematic approach for extended sources, with a maximum peak ground velocity value of 1 Hz. The qualitative and quantitative comparison of these simulations with the macroseismic intensity database allows us to discriminate between various sources and configurations. The quantitative validation of the seismic source is done using ad hoc ground motion to intensity conversion equations (GMICEs), expressly calculated for this study. This study allows us to identify the most probable causative source model of this event, contributing to the improvement of the seismotectonic knowledge of this region. The candidate fault that has the lowest values of average differences between observed and calculated intensities and chi-squared is a strike slip fault with a toward-north rupture as the Ortnek fault.

**Electronic supplementary material:**

The online version of this article (10.1007/s10950-018-9743-z) contains supplementary material, which is available to authorized users.

## Introduction

**Fig. 1:**
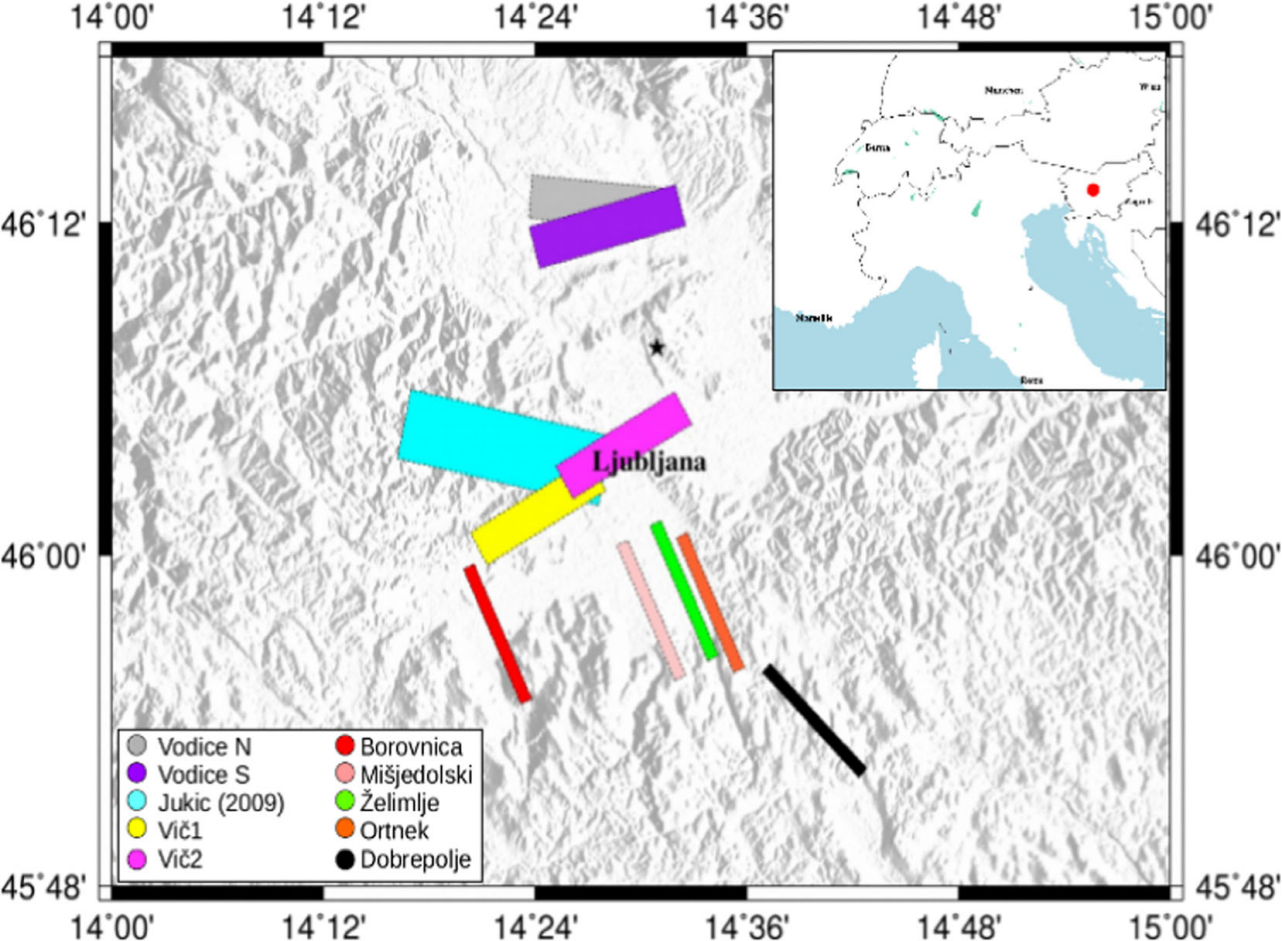
Map of the studied faults in this work. The five strike-slip faults to the south of Ljubljana are as follows: the red, the Borovnica fault; the pink one, the Mišjedolski; the green one, the Želimlje fault; the orange, the Ortnek; the black one, the Dobrepolje. The Vič fault is divided into two segments: the yellow one (Vič-a) is the first segment used in the Vič 1–2 simulations, the magenta one is the second segment (Vič-b) of the Vič fault for the 3–4 simulations. The light blue fault is the inversion result of the Jukić (2009) study; the gray and the purple ones are respectively the north segment and the south segment of the Vodice fault. The black star is the macroseismic epicenter from the SHEEC catalog. In the small map, the red point is the Ljubljana location

The 1895 earthquake occurred in the surroundings of Ljubljana, the capital of Slovenia, presently with more than 300,000 inhabitants (Fig. 1), and then the capital of Austro-Hungarian crown land Carniola.

The knowledge of the historical and pre-instrumental events that occurred in this area is fundamental for the study of the hazard of the area. In particular, the 1895 earthquake is the strongest historical event of the central Slovenia region, so the identification of its seismogenic source is crucial for this densely populated area, in order to mitigate possible future earthquake-related damage.

The only available information about historical earthquakes comes from the macroseismic intensity data points, from which it is possible to derive an estimated value of the moment magnitude and a hypothetical location of the event. This information can be used to estimate “synthetic” intensities from peak ground velocity (PGV) values. In this study, the PGV values are obtained using two different techniques: simply retrieving them from existing GMPEs (Massa et al. 2008; Akkar and Bommer 2010; Bindi et al. 2014; Cauzzi et al. 2014) based on the moment magnitude value and event epicenter; or by computing synthetic PGV ground motion scenarios for a given fault model. Taking into account all the well-known faults surrounding the hypothetical epicenter and the estimated moment magnitude value, we can compare the PGV-derived intensities with the observed ones in order to discriminate which of the studied faults is the most probable causative one.

In order to compare intensities, it is necessary to find a relationship between the observed intensities and the PGV obtained from the computed synthetic seismograms and the GMPEs. The GMICE that has been estimated for this purpose differs from the other ones found in literature (e.g., Faccioli and Cauzzi 2006; Faenza and Michelini 2010) for both the quantity and quality of the near-fault accelerometric data used. The data are taken from the CE3RN-Central Eastern European Earthquake and Research Network (Costa et al. 2010; Bragato et al. 2014) and the Italian RAN-Rete Accelerometrica Nazionale databases (Gorini et al. 2010; Costa et al. 2015). The upper frequency limit for the ground motion scenarios is 1 Hz; so, in order to be consistent with the maximum frequency of the synthetic seismograms, another GMICE is estimated using 1 Hz as the frequency limit. The main advantage of working directly with waveforms is the possibility to low-pass filter the observed records.

The main goal of this work is to apply this relationship to analyze various possible scenarios for the Ljubljana 1895 event and to identify, by comparing observed and PGV-based intensities, its most probable causative fault. The studied faults are the nearest ones to the Ljubljana city: Vič, Želimlje, Borovnica, Vodice, Ortnek, Mišjedolski, and Dobrepolje faults, as well as a fault proposed by an inversion work (Jukić 2009) (Fig. 1). The PGV is estimated using different GMPEs found in literature (Massa et al. 2008; Akkar and Bommer 2010; Bindi et al. 2014; Cauzzi et al. 2014). When computing the PGV1Hz, more than one configuration (different positions of the nucleation point) is used to produce the ground motion scenarios for each considered fault. All the set of PGV and PGV1Hz (with a maximum frequency of 1 Hz), calculated at localities with observed intensities, are converted into PGV-derived intensities using the new GMICEs, and then compared with the observed intensities. The lowest overall misfit identifies the most probable causative fault and related scenario.

## Tectonic summary

**Fig. 2:**
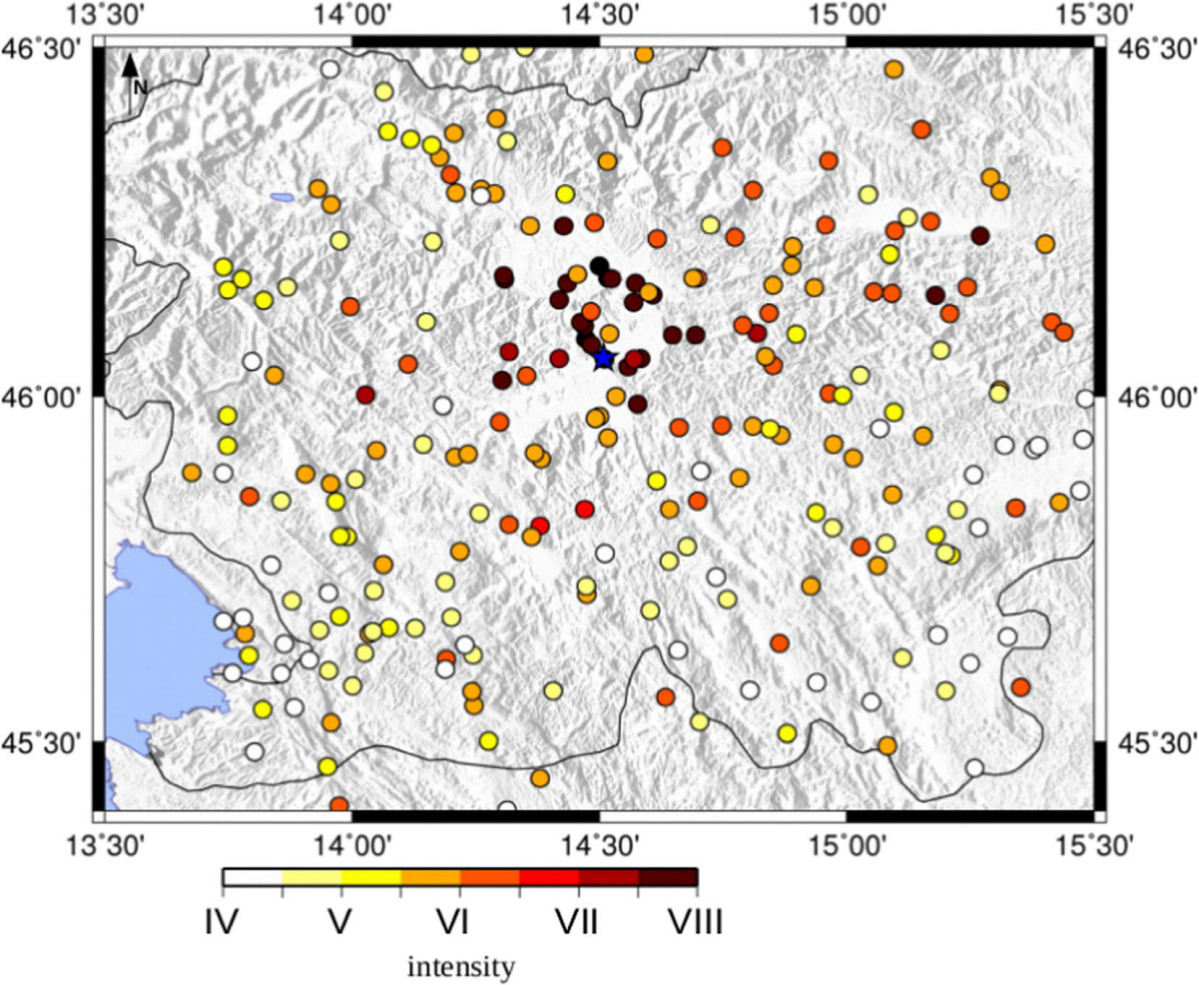
The 1895 Ljubljana intensity data points from Cecić (1998); the blue star is the Ljubljana location

**Fig. 3:**
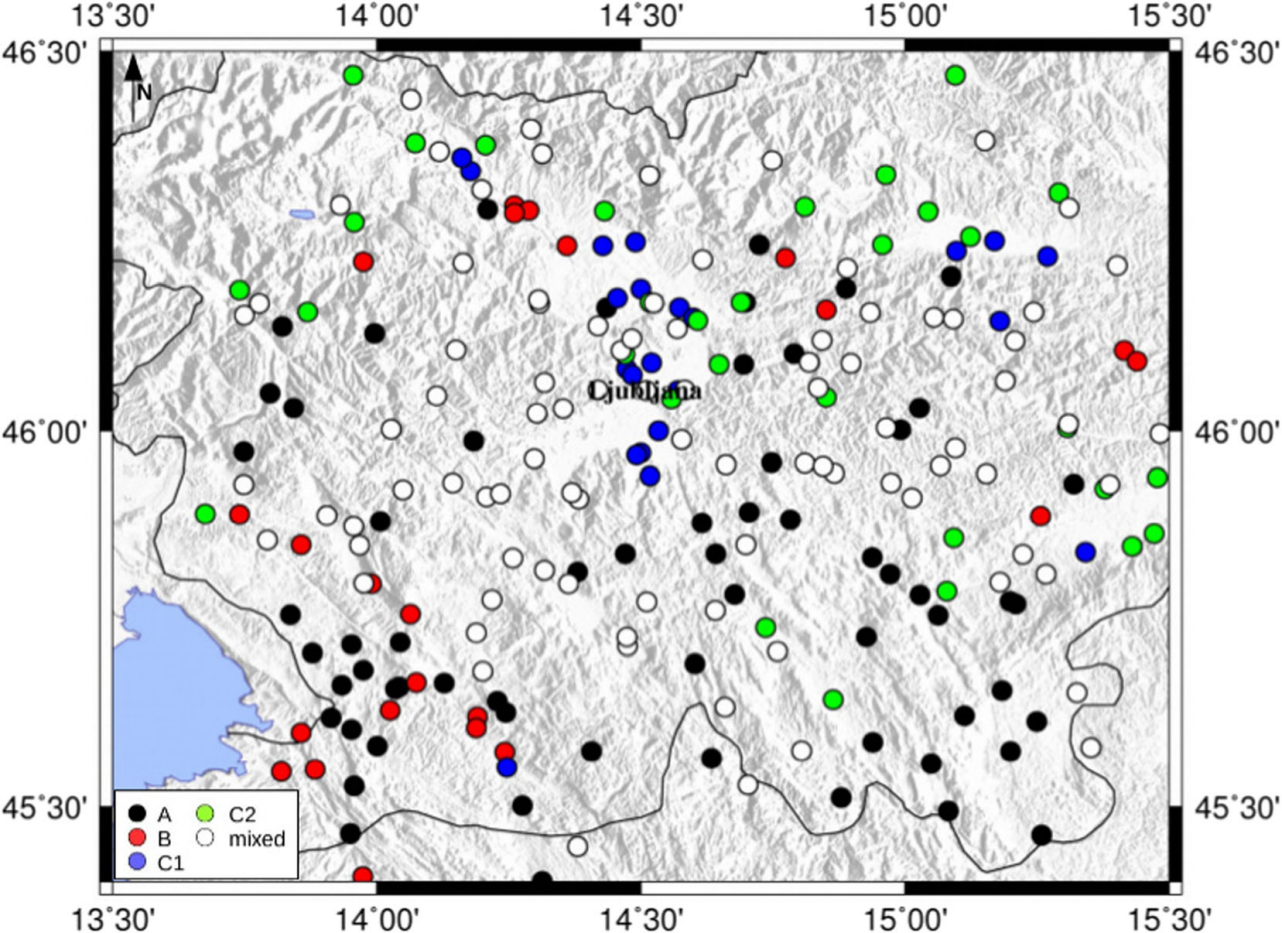
Distribution of the site classification, proposed by Sirovich et al. (2012), in the observed intensity points for the 1895 Ljubljana event. A, solid rock; B, stiff, soft rocks and very dense soils; C1, soft sediments with thickness over 30 m; C2, soft sediments with thickness of 5–30 m; mixed when more than one type was present

The Ljubljana earthquake, that led to the first seismological observations in the former Austro-Hungarian monarchy, occurred on April 14, 1895. The related macroseismic intensity dataset comes from the ARSO macroseismic archive (Cecić 1998) (see Fig. 2). It consists of 801 intensity data points (IDPs) with the intensities from VIII-IX to I EMS-98. The maximum intensity VIII-IX EMS-98 was observed in four localities (Ljubljana, Dravlje, Utik, Vodice). In this study, only points taken into account are far at least 90 km from the macroseismic epicenter (Cecić 1998) (see Fig. 2). This is to allow in the computation only direct S-waves as the ones carrying the highest amplitudes and avoid distances at which the Moho-reflected wave takes over as the ones carrying the highest amplitude, distorting the source radiation pattern and thus our analysis. Its moment magnitude estimate, as reported by the SHARE European catalog (SHEEC), is 5.9 according to Cecić (1998), while in Živčić (2009) is 6.1. The last estimation is 6.0 from CPTI15, consistent with the previous ones, so used in this study. All these reported magnitude values are rounded to one decimal value only. The maximum intensity was observed in the Ljubljana surroundings, with a value VIII-IX (EMS-98). The highest values are located in the Ljubljana Basin, north of the city, and in the northern part of the city itself, probably due to the site effects of amplification of the basin (Sirovich et al. 2012; Gosar et al. 2010). Different soil types at locations of observed intensities represented in Fig. 3 are taken from the work of Sirovich et al. (2012). Areas with C1 type (soft sediments with thickness over 30 m) are the most prone to amplifications of earthquake ground motion, followed by the areas with C2 type (soft sediments with thickness bellow 30 m) (Sirovich et al. 2012).

The possible causative faults considered in this study are summarized in Fig. 1, where the surface projections are shown: Vič, Borovnica, Mišjedolski, Želimlje, Ortnek, Dobrepolje, and Vodice faults and a result of an inversion work (Jukić 2009).

The latter one is obtained inverting the intensity macroseismic data points of the Ljubljana event and has a WNW-ESE orientation, with a strike angle of 282°. The direction of the slip vector, assuming a thrust mechanism, is 86°, whereas the fault dip is about 38°. This theoretical solution, however, cannot be linked to any of the known faults at the surface.

The other faults, candidates for the forward modeling, are selected due to their proximity to Ljubljana City and their seismogenic capacity.

The Vič fault is a 235°–250° striking fault at the N boundary of the Ljubljana Moor. It runs between the Drenov Grič village on W and the Sava River on E (Grad and Ferjančič 1974; Premru 1982; Verbič 2006). The Vič fault has been interpreted as a reverse northward dipping fault exhibiting Quaternary activity (Verbič 2006). Its dip is estimated at 45°–80° N. Given the fault orientation and a N-S directed recent compression, a minor sinistral component of motion is considered in addition to the pure reverse one (rake estimation between 60° and 90°). This fault was “segmented” to have two shorter segments compatible with the 10-km-long structure (referring to the magnitude of the event): its easternmost and westernmost parts.

South of the Ljubljana Moor there is a set of a parallel NW–SE striking dextral faults with similar characteristics, belonging to the Dinaric Fault System (the common name for NW–SE striking dextral neotectonic faults in the area; see, for example, Moulin et al. 2016): Borovnica, Mišjedolski, Želimlje, Ortnek, and Dobrepolje faults.

The Borovnica fault runs near the western boundary of the Ljubljana Moor and extends further toward SE (Buser et al. 1967; Placer et al. 2010). Based on geological mapping, the fault has a strike angle of 300°–330° and is dipping between 70° and 85° NE (Buser et al. 1967; Buser 1970).

The Mišjedolski fault is a steep, almost vertical, NE dipping dextral strike-slip fault running from the town of Ig along Mokrec Hill, through the Mišja dolina (Mišja Valley) further toward SE (Buser 1969, 1974). Its strike is measured to be 320°–340° and its dip is estimated on 70°–85°.

The Želimlje fault is mapped as a dextral strike-slip fault with a strike between 320° and 345°, running from the eastern part of the Ljubljana Moor through Želimlje Valley toward Ortnek, further to Žlebič, where it merges with the parallel Ortnek fault. Its dip is estimated on 70°–85° (Buser 1969, 1974).

Ortnek fault runs from the Ljubljana Moor through Pijava Gorica, Smrjene, and Rogatec and east of Rašica, Velike Lašče, and Ortnek (Buser 1969, 1974). From here on, it continues toward SE through Ribnica and Kočevje polje (Buser 1974). Based on structural geological mapping, this is a 320°–340° striking fault with 75°–80° dip toward NE.

Dobrepolje fault is the easternmost one from the series of dextral strike-slip faults S of the Ljubljana Moor (Buser 1969, 1974). This fault has a strike of 290°–340° and a dip of 70°–85° toward NE.

The displacements along all five dextral faults are almost pure dextral with some inherited normal component from the previous phases of activity (cf. Vrabec and Fodor 2006). Given the fault orientations in recent N-S directed compression, a minor reverse component of motion is expected (similar as in Basili et al. 2013). The rake is therefore estimated to be 160°–180° for Borovnica, Mišjedolski, Želimlje, Ortnek, and Dobrepolje faults.

North of Ljubljana, there is another candidate for the seismic source. The reverse Vodice fault runs between Sava and Pšata Rivers and is exhibited in the surface with two fault scarps, each presenting one segment (Verbič 2006; Jamšek Rupnik et al. 2013). Strike, dip, and rake of the fault are estimated from geomorphological mapping, structural-geological data, and geophysical data (Jamšek Rupnik 2013). The estimated values for the N segment are as follows: strike 275°, dip 55°, and rake 90°. The estimated values for the S segment are as follows: strike 255°, dip 55°, and rake 90°. Despite that the macroseismic intensity dataset of the Ljubljana 1895 earthquake does not seem to fit with intensities expected on such fault (maximum intensities should be observed on the hanging wall, thus N of the fault trace), we choose to simulate also the two segments of the Vodice fault as a possible generating fault, in order to explore all known nearby faults as the possible generators of this event.

The source mechanisms of the eight studied faults are summarized in Table S1.

## GMICE

In the last years, the many records from new strong events and a number of newly installed accelerometric stations and from many quality-upgraded old ones made the previously estimated GMICEs found in literature (e.g., Faenza and Michelini 2010; Caprio et al. 2015) outdated. In this work, the quality and the quantity of the recent near-fault accelerometric data used provide more reliable GMICEs with respect to the previous ones. We estimate a classical GMICE and another one using the upper frequency limit 1 Hz. The first considers the full recorded frequency range and it will be used to convert the GMPE-derived PGV values into estimated PGV intensity values.

On the other hand, the need of a 1-Hz GMICE is demanded by the upper frequency limit at 1 Hz of the synthetic seismograms calculated in this work. The choice of this frequency limit depends on the fact that at high frequencies the waveforms are strongly influenced by the unknown complexity of the medium through which the waves propagate. In this work, a 1D velocity model is used and it is adequate for 1-Hz maximum frequency simulations (Fitzko et al. 2004, 2005; Tiberi et al. 2014).

Table S2 reports the 24 events (Mw > 4) in the time span 2002–2016, used in this work, taken from the RAN and CE3RN databases. The database construction and the processing of data, such as the phase picking, the event locations, and the local magnitude estimation, are done using Antelope® (BRTT, Boulder). The macroseismic intensity data points for the Slovenian events are taken from the Slovenian Environment Agency (ARSO) macroseismic archive (ARSO 2012), whereas the Italian data come from the Italian Macroseismic Databases (DBMI11 and DBMI15). The Slovenian intensity values are in EMS-98 scale, while the Italian ones are in MCS scale. Described as well in Musson et al. (2010), the only difference between the MCS and EMS-98 scales is the saturation point of the EMS-98 at intensity of 11 while the MCS one is at 12. In this study, the last classes of intensity are not used so it is possible to use these two scales indiscriminately. The PGV values are computed from the recorded accelerometric waveforms using the automatic procedure implemented in Antelope® by the SeisRaM group at the Department of Mathematics and Geosciences (DMG) of the University of Trieste (Gallo et al. 2014).

The maximum PGV value between the two horizontal component values is defined as the maximum observed PGV in order to have a consistent comparison with the maximum PGV values calculated in the simulations.

The criterion for the association of the PGV value calculated at each station site with an intensity point of the related macroseismic dataset is the minimum distance between the two sites. In this work, the maximum distance value between the 115 PGV intensity pairs is 5 km, the mean value being 1.8 ± 1.8 km.

The PGV-derived intensity data points are grouped into half-integer classes of interval, in order to be consistent with the observed intensity values. For the estimation of the GMICE, the data are binned into classes of half-integer intensity, and for each class the geometrical mean *μ*_g_ is calculated as follows:

1 $$ {\mu}_{\mathrm{g}}={\Sigma}_i{\log}_{10}{\mathrm{PGV}}_i/n $$

where *n* is the total number of PGV_*i*_ for each intensity class. The associated geometrical standard deviation is:

2 $$ {\sigma}_{\mathrm{g}}=\exp \left[\surd {\Sigma}_i{\left({\log}_{10}{\mathrm{PGV}}_i\hbox{-} {\mu}_{\mathrm{g}}\right)}^2/n\ \right] $$

The data are fitted using a linear relationship between the intensity *I* and the base 10 logarithm of the PGV:

3 $$ I=a\times {\log}_{10}\mathrm{PGV}+b $$

The methodology used for the data fitting is the non-linear least-squares (NLLS) Marquardt-Levenberg algorithm (Levenberg 1944), that is an iterative technique that calculates the minimum of a function expressed as a sum of squares of non-linear functions. Applying this algorithm to our filtered dataset at 1 and 80 Hz, the obtained relationships are, respectively:

4 $$ I=\left(1.46\pm 0.18\right)\times {\log}_{10}\mathrm{PGV}1\mathrm{Hz}+\left(5.62\pm 0.20\right) $$

5 $$ I=\left(1.84\pm 0.24\right)\times {\log}_{10}\mathrm{PGV}+\left(4.85\pm 0.23\right) $$

**Fig. 4:**
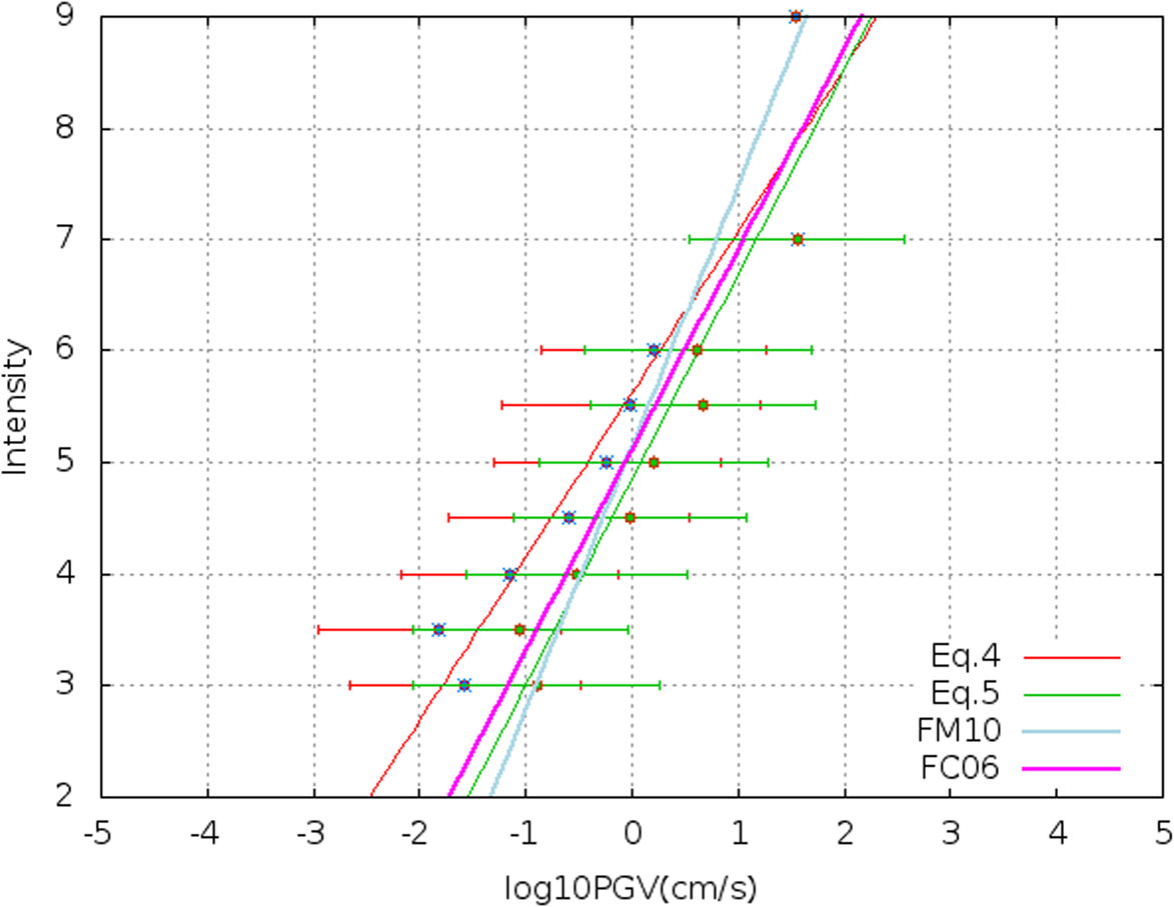
Intensity versus PGV1Hz in red and PGV in green. The blue and red stars are the geometrical mean for each intensity class and the red and green lines are the associated geometrical standard deviation. The light blue and the magenta lines are respectively the Faenza and Michelini (2010) and Faccioli and Cauzzi (2006) GMICEs

The resulting GMICEs are shown in Fig. 4. The associated coefficient of determination, the *R*^2^ value, for Eq. 4 is 0.90 and the related standard deviation is 0.60. For Eq. 5, the *R*^2^ value is 0.89 and the standard deviation 0.66.

## Use of empirical GMPEs and GMICEs

In order to use GMPEs to derive PGV values that will be used to estimate intensities, we need to establish the fault model and related nucleation point in order to compute the related distances. The possible fault and rupturing models of the Ljubljana earthquake are chosen to be the same as those of the candidate faults that will be used in the forward scenario modeling (see next section).

For each fault model (Fig. 1), the PGV values are calculated using various ground motion prediction equations found in literature (Massa et al. 2008; Akkar and Bommer 2010; Bindi et al. 2014; Cauzzi et al. 2014). The Massa et al. (2008) equation depends on the epicentral distance and soil classification (two classes: rock or soft soil). The site classification of the observed intensity localities is shown in Fig. 3, taken from the work of Sirovich et al. (2012). The conversion between the classes in Sirovich et al. (2012) and the soil classification coefficients in each GMPE are reported in Table S3. Akkar and Bommer (2010) uses the Joyner and Boore distance, Rjb (Joyner and Boore 1993), two site classes as in Massa et al. (2008), and a fault mechanism dependent coefficient for normal or reverse fault. Bindi et al. (2014) uses the Rjb distance, the four EC8 site classes (A–D), and three different fault classes: normal, reverse, and strike-slip. The Cauzzi et al. (2014) GMPE basically differs from that of the Bindi et al. (2014) from the distance used, the distance to the closest point on the rupture plane distance (Rrup) defined in Kaklamanos et al. (2011).

The PGV values, obtained using Mw = 6.0, are converted into estimated intensities using our GMICE, Eq. 5 (Table 1), the Faenza and Michelini (2010) law (Table 2), and the revision of this latter one made in Caprio et al. (2015) in Table 3. The results in terms of intensity differences (*I*_observed_ – *I*_calculated_) and the misfit values, *Σ*_*i*_ |*I*_observed_ – *I*_calculated_|_*i*_, are reported with the correspondent variance values in intervals of half-integer intensity in order to be consistent with the integer observed intensity values. It is evident that it is not possible to discriminate which of the fault models give the best fit. This could be due to the fact that all the sources are relatively close to each other, and the different fault term is not relevant in the estimation of the final PGV value. We also note that the Faenza and Michelini (2010) GMICE produces average intensity differences and misfit values higher than those calculated with our proposed GMICE (Eq. 5). Otherwise, the results obtained using the Caprio et al. (2015) GMICE adding the Italian regional coefficient to the global GMICE are really similar to those obtained with the Eq. 5, so this will lead to the same conclusions.

**Table 1:** The mean values of the intensity differences and the misfit values with the correspondent variance value, for each GMPE used and for each studied fault, are reported using Eq. 5 GMICE

	Massa et al. (2008)	Akkar and Bommer (2010)	Bindi et al. (2014)	Cauzzi et al. (2014)
Fault	Intensity differences mean value	Intensity differences mean value	Intensity differences mean value	Intensity differences mean value
All faults	0.0 ± 0.5	−1.0 ± 0.5	−1.5 ± 0.5	0.5 ± 0.5
	Misfit value	Misfit value	Misfit value	Misfit value
Vič 1	176.5 ± 0.5	305.0 ± 0.5	489.0 ± 0.5	185.5 ± 0.5
Vič 2	164.5 ± 0.5	300.5 ± 0.5	490.0 ± 0.5	172.5 ± 0.5
Želimlje	183.5 ± 0.5	298.5 ± 0.5	402.0 ± 0.5	188.5 ± 0.5
Borovnica	196.5 ± 0.5	304.5 ± 0.5	400.0 ± 0.5	203.5 ± 0.5
Jukić(2009)	176.0 ± 0.5	310.0 ± 0.5	497.5 ± 0.5	188.0 ± 0.5
Vodice N	161.5 ± 0.5	286.0 ± 0.5	470.0 ± 0.5	185.0 ± 0.5
Vodice S	159.5 ± 0.5	289.0 ± 0.5	471.5 ± 0.5	171.0 ± 0.5
Ortnek	179.5 ± 0.5	299.0 ± 0.5	402.5 ± 0.5	181.5 ± 0.5
Mišjedolski	180.0 ± 0.5	299.5 ± 0.5	402.0 ± 0.5	181.5 ± 0.5
Dobropolje	195.5 ± 0.5	306.5 ± 0.5	405.5 ± 0.5	193.0 ± 0.5

**Table 2:** The mean values of the intensity differences and the misfit values with the correspondent variance value, for each GMPE used and for each studied fault, are reported using the Faenza and Michelini (2010) GMICE

	Massa et al. (2008)	Akkar and Bommer (2010)	Bindi et al. (2014)	Cauzzi et al. (2014)
Fault	Intensity differences mean value	Intensity differences mean value	Intensity differences mean value	Intensity differences mean value
Vič 1	0.0 ± 0.5	− 2.0 ± 0.5	− 2.5 ± 0.5	0.0 ± 0.5
Vič 2	− 0.5 ± 0.5	− 1.5 ± 0.5	− 2.5 ± 0.5	0.0 ± 0.5
Želimlje	− 0.5 ± 0.5	− 2.0 ± 0.5	− 2.5 ± 0.5	0.0 ± 0.5
Borovnica	− 0.5 ± 0.5	− 2.0 ± 0.5	− 3.0 ± 0.5	0.0 ± 0.5
Jukić(2009)	− 0.5 ± 0.5	− 2.0 ± 0.5	− 2.5 ± 0.5	0.0 ± 0.5
Vodice N	− 0.5 ± 0.5	− 2.0 ± 0.5	− 2.5 ± 0.5	0.0 ± 0.5
Vodice S	− 0.5 ± 0.5	− 2.0 ± 0.5	− 3.0 ± 0.5	0.0 ± 0.5
Ortnek	− 0.5 ± 0.5	− 2.0 ± 0.5	− 3.0 ± 0.5	0.0 ± 0.5
Mišjedolski	− 0.5 ± 0.5	− 2.0 ± 0.5	− 3.0 ± 0.5	0.0 ± 0.5
Dobropolje	− 0.5 ± 0.5	− 2.0 ± 0.5	− 3.0 ± 0.5	0.0 ± 0.5
	Misfit value	Misfit value	Misfit value	Misfit value
Vič 1	176.5 ± 0.5	452.0 ± 0.5	719.0 ± 0.5	195.5 ± 0.5
Vič 2	212.5 ± 0.5	453.5 ± 0.5	721.5 ± 0.5	180.0 ± 0.5
Želimlje	223.0 ± 0.5	440.0 ± 0.5	602.0 ± 0.5	190.5 ± 0.5
Borovnica	235.0 ± 0.5	435.0 ± 0.5	591.5 ± 0.5	207.5 ± 0.5
Jukić(2009)	216.0 ± 0.5	462.5 ± 0.5	730.5 ± 0.5	199.5 ± 0.5
Vodice N	192.5 ± 0.5	430.0 ± 0.5	695.0 ± 0.5	184.5 ± 0.5
Vodice S	194.5 ± 0.5	435.5 ± 0.5	701.5 ± 0.5	178.5 ± 0.5
Ortnek	220.5 ± 0.5	439.5.0 ± 0.5	602.5 ± 0.5	185.0 ± 0.5
Mišjedolski	221.5 ± 0.5	439.0 ± 0.5	602.0 ± 0.5	185.0 ± 0.5
Dobropolje	230.5 ± 0.5	441.0 ± 0.5	597.0 ± 0.5	194.5 ± 0.5

**Table 3:** The mean values of the intensity differences and the misfit values with the correspondent variance value, for each GMPE used and for each studied fault, are reported using the Caprio et al. (2015) GMICE

Fault	Intensity differences mean value	Intensity differences mean value	Intensity differences mean value	Intensity differences mean value
All Faults	0.0 ± 0.5	− 1.0 ± 0.5	− 1.5 ± 0.5	0.5 ± 0.5
	Misfit value	Misfit value	Misfit value	Misfit value
Vič 1	218.5 ± 0.5	282.0 ± 0.5	427.5 ± 0.5	180.5 ± 0.5
Vič 2	164.0 ± 0.5	277.5 ± 0.5	427.0 ± 0.5	168.0 ± 0.5
Želimlje	181.5 ± 0.5	277.0 ± 0.5	358.5 ± 0.5	183.0 ± 0.5
Borovnica	193.5 ± 0.5	283.0 ± 0.5	357.0 ± 0.5	197.0 ± 0.5
Jukić(2009)	175.0 ± 0.5	286.0 ± 0.5	434.5 ± 0.5	181.5 ± 0.5
Vodice N	162.0 ± 0.5	266.0 ± 0.5	411.0 ± 0.5	177.0 ± 0.5
Vodice S	160.0 ± 0.5	268.0 ± 0.5	414.5 ± 0.5	172.0 ± 0.5
Ortnek	178.0 ± 0.5	277.5 ± 0.5	359.0 ± 0.5	177.5 ± 0.5
Mišjedolski	178.5 ± 0.5	278.0 ± 0.5	358.5 ± 0.5	177.5 ± 0.5
Dobropolje	193.0 ± 0.5	284.5 ± 0.5	364.0 ± 0.5	187.0 ± 0.5

## Ground motion scenarios

The synthetic seismograms obtained from the simulations are calculated using the multi-modal summation technique (Panza and Suhadolc 1987; Fitzko et al. 2004) with a kinematic approach of the source (Saraò et al. 1998), as described in Tiberi et al. (2014). This calculation requires as input data the following: the source parameters and a 1D velocity model. The choice of the upper frequency limit of the synthetics is fundamental for the reliable estimation of ground motion parameters. In fact, the high frequencies are strongly influenced by the (unknown) small wavelength velocity structure. The peak ground velocity is therefore estimated with a maximum frequency of 1 Hz using a 1D velocity model “est4a” proposed by Costa et al. (1992, 1993). The PGV1Hz values are calculated at the same localities where the intensity estimation was done, the intensity data points being illustrated in Fig. 2.

The source configurations used in this work for the various ground motion scenarios are summarized in Table S4.

For the Vič fault simulations, two different segments of the entire fault are analyzed: the first two scenarios are computed using a part of the fault to the SE of Ljubljana (Vič 1–2); the second two using a part immediately to the north of Ljubljana (Vič 3–4). All the scenarios related to the same fault differ only for the nucleation point, shown in the last column of Table S4.

The first set of simulations are computed using as moment magnitude the value Mw = 6 proposed by SHEEC (Živčić 2009) equivalent to a total seismic moment *M*_0_ = 1.0 + 25 dyne × cm (Kanamori 1977). For each site/point of the simulation, the maximum value of the PGV1Hz taken from the horizontal components is considered, and the respective intensity is calculated using the GMICE proposed in the first part of this study.

In Table 4, the mean values of the intensity differences and the misfit values are reported as in Table 1 for the GMPE equation application. In this case, the minima of these averages of differences and of the misfit values are obtained using the strike-slip faults to the south of Ljubljana as causative faults, with a nucleation point positioned at the southern tip of the fault.

**Table 4:** In this table, the mean values of the intensity differences for each scenario and the misfit values with the correspondent variance value are reported, for the simulations with Mw = 6 and 5.4

6 Mw			5.4 Mw	
Fault	Intensity differences mean value	Misfit value	Intensity differences mean value	Misfit values
Vič 1	− 1.5 ± 0.5	363.0 ± 0.5	− 0.0 ± 0.5	190.0 ± 0.5
Vič 2	− 2.0 ± 0.5	497.5 ± 0.5	− 0.5 ± 0.5	249.0 ± 0.5
Vič 3	− 1.5 ± 0.5	362.0 ± 0.5	−0.0 ± 0.5	188.5 ± 0.5
Vič 4	− 2.0 ± 0.5	498.0 ± 0.5	−1.0 ± 0.5	246.0 ± 0.5
Želimlje 1	− 1.0 ± 0.5	295.5 ± 0.5	*0.0 ± 0.5*	*182.5 ± 0.5*
Želimlje 2	− 1.0 ± 0.5	293.5 ± 0.5	0.0 ± 0.5	184.5 ± 0.5
Borovnica 1	− 1.0 ± 0.5	305.5 ± 0.5	0.0 ± 0.5	194.0 ± 0.5
Borovnica 2	− 1.0 ± 0.5	290.5 ± 0.5	0.5 ± 0.5	192.5 ± 0.5
Jukić(2009)	− 2.0 ± 0.5	457.0 ± 0.5	−0.0 ± 0.5	209.5 ± 0.5
Vodice N	− 2.0 ± 0.5	459.0 ± 0.5	−0.5 ± 0.5	223.5 ± 0.5
Vodice S	− 2.0 ± 0.5	485.5 ± 0.5	−0.5 ± 0.5	241.5 ± 0.5
Ortnek	− 1.0 ± 0.5	298.5 ± 0.5	*0.0 ± 0.5*	*181.5 ± 0.5*
Mišjedolski	− 1.0 ± 0.5	293.0 ± 0.5	0.0 ± 0.5	185.0 ± 0.5
Dobropolje	− 1.0 ± 0.5	320.5 ± 0.5	0.0 ± 0.5	190.5 ± 0.5

The italicized values are the best combination in terms of intensity differences mean value and misfit values, found using as causative faults the Želimlje 1 and Ortnek faults

It is possible to notice that the mean values are negative; this means that the synthetic PGV1Hz values are overestimated with respect to all the observed intensity data points. For that reason, in order to obtain the set of computed intensity values, with a zero mean value of differences between the observed intensity points, a set of ground motion scenarios is computed using decreasing Mw values; in Table 4, the results for the (Mw = 5.4) simulations are reported. It is possible to notice that the minimum of the average differences is 0 and the lowest misfit (Table 4) is obtained considering as generating fault, as for the (Mw = 6) scenarios, one of the strike-slip faults with a toward-north rupture.

**Fig. 5:**
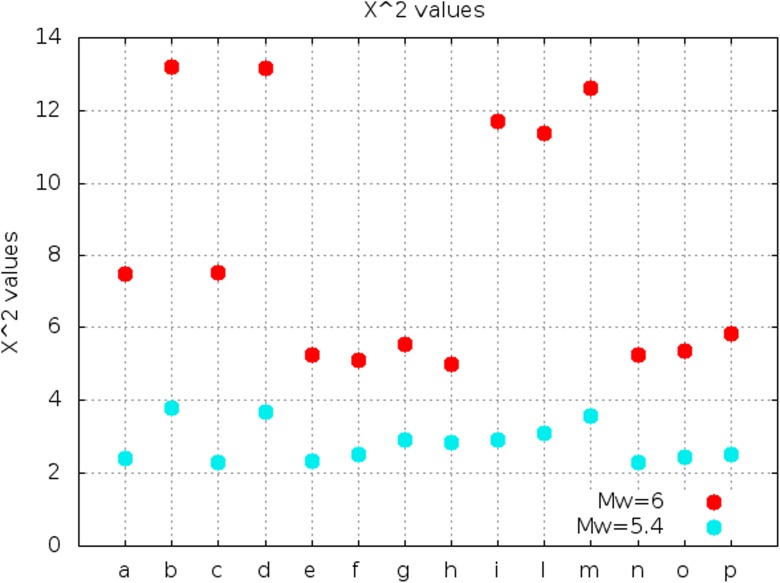
Chi-squared values using different moment magnitudes. a is the simulation Vič1; b, Vič2; c, Vič3; d, Vič4; e, Želimlje1; f, Želimlje2; g, Borovnica1; h, Borovnica2; i, Jukić; l, Vodice N; m, Vodice S; n, Ortnek; o, Mišjedolski; and p, Dobrepolje The “closest to one” value of all these points is obtained by the simulation of the Ortnek fault with a nucleation point at the south of the fault with a moment magnitude of 5.4

In order to test the reliability of the calculated intensities with respect to the observed ones using another methodology, the estimation of the chi-squared, reduced for the degrees of freedom, is reported in Table 5 and Fig. 5 for each scenario (described in Table S4). The “closest to one” value is the one calculated for the first scenario of the Ortnek fault with a moment magnitude of 5.4, corresponding to the minimum value of all mean differences and misfit values. All these criteria, therefore, point toward a strike-slip fault with a toward-north rupture and a moment magnitude of 5.4, as the most probable one to have generated the Ljubljana earthquake.

**Table 5:** Summary of the chi-squared values for each simulation calculated with all the moment magnitude values analyzed

	6.0 Mw	5.4 Mw
Fault	*X* ^2^	*X* ^2^
Vič 1	7.48	2.41
Vič 2	13.20	3.79
Vič 3	7.51	2.29
Vič 4	13.16	3.68
Želimlje 1	5.26	2.33
Želimlje 2	5.12	2.50
Borovnica 1	5.53	2.91
Borovnica 2	5.00	2.86
Jukić (2009)	11.71	2.92
Vodice N	11.38	3.11
Vodice S	12.63	3.56
Ortnek	5.26	2.28
Mišjedolski	5.35	2.45
Dobropolje	5.82	2.52

Another possible way to find the most probable generating fault could be trying to understand if the scenario-generated synthetic PGV1Hz values are consistent with the obtained and used GMICE, previously described in this work. For this purpose, the 15 obtained synthetic sets of PGV1Hz values are used in order to estimate the related synthetic GMICEs. After obtaining the 15 synthetic laws, we have checked if the GMICE obtained from the observed data is compatible with any of them. The observed GMICE falls within the standard deviation of the data of all the synthetic laws, so this criterion cannot be used to identify the causative fault. In fact, all the synthetic PGV1Hz values are comparable with respect to the observed ones (Fig. S1).

**Fig. 6:**
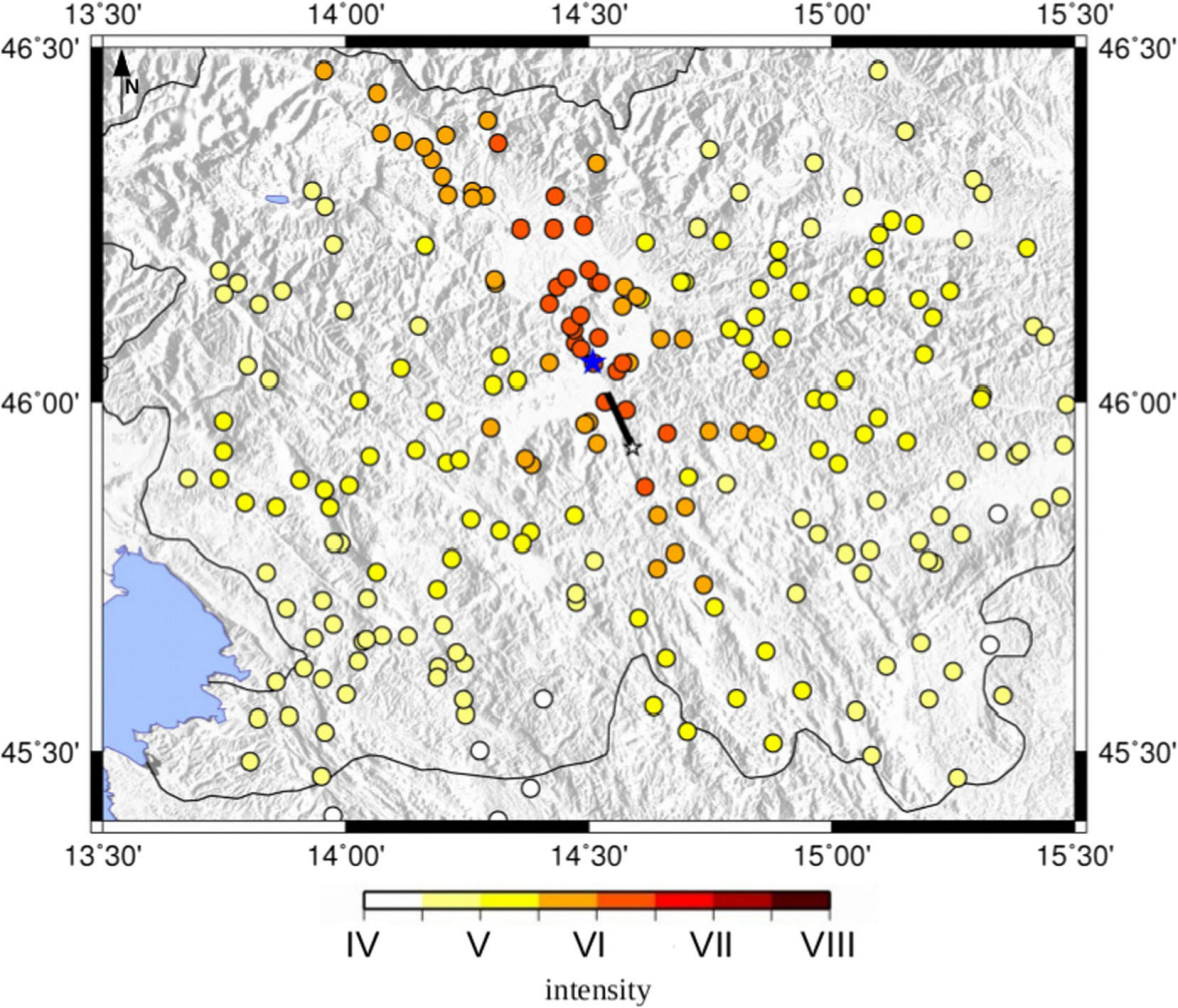
Calculated intensity data points for the 1895 Ljubljana event simulated with the most probable causative fault: the Ortnek one with a nucleation point at the south of the fault; the blue star is the Ljubljana location

**Fig. 7:**
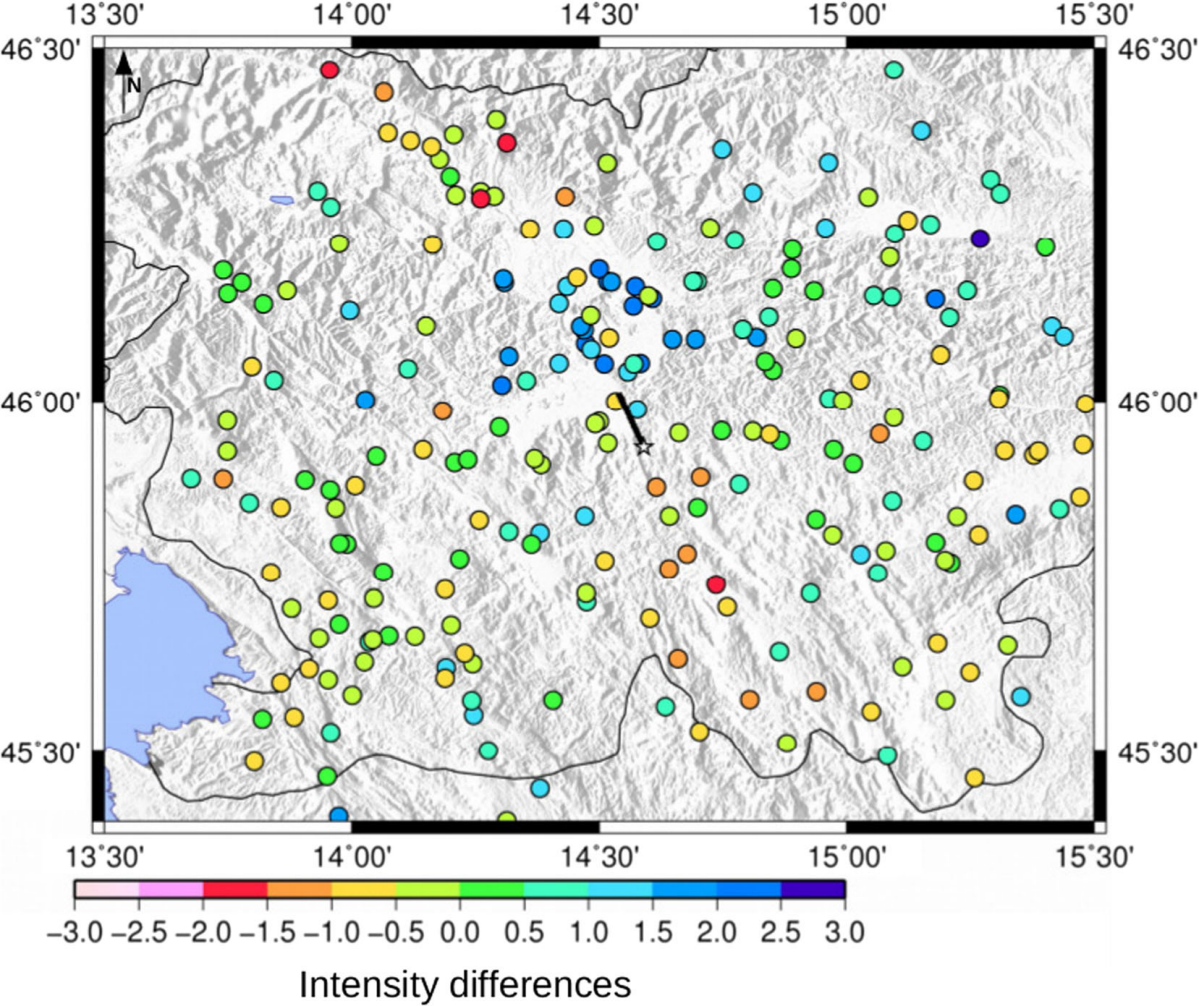
Map reporting the distribution of the intensity differences (*I*_observed_ − *I*_calculated_) for the scenario using as generating fault the Ortnek one with a toward-north rupture

**Fig. 8:**
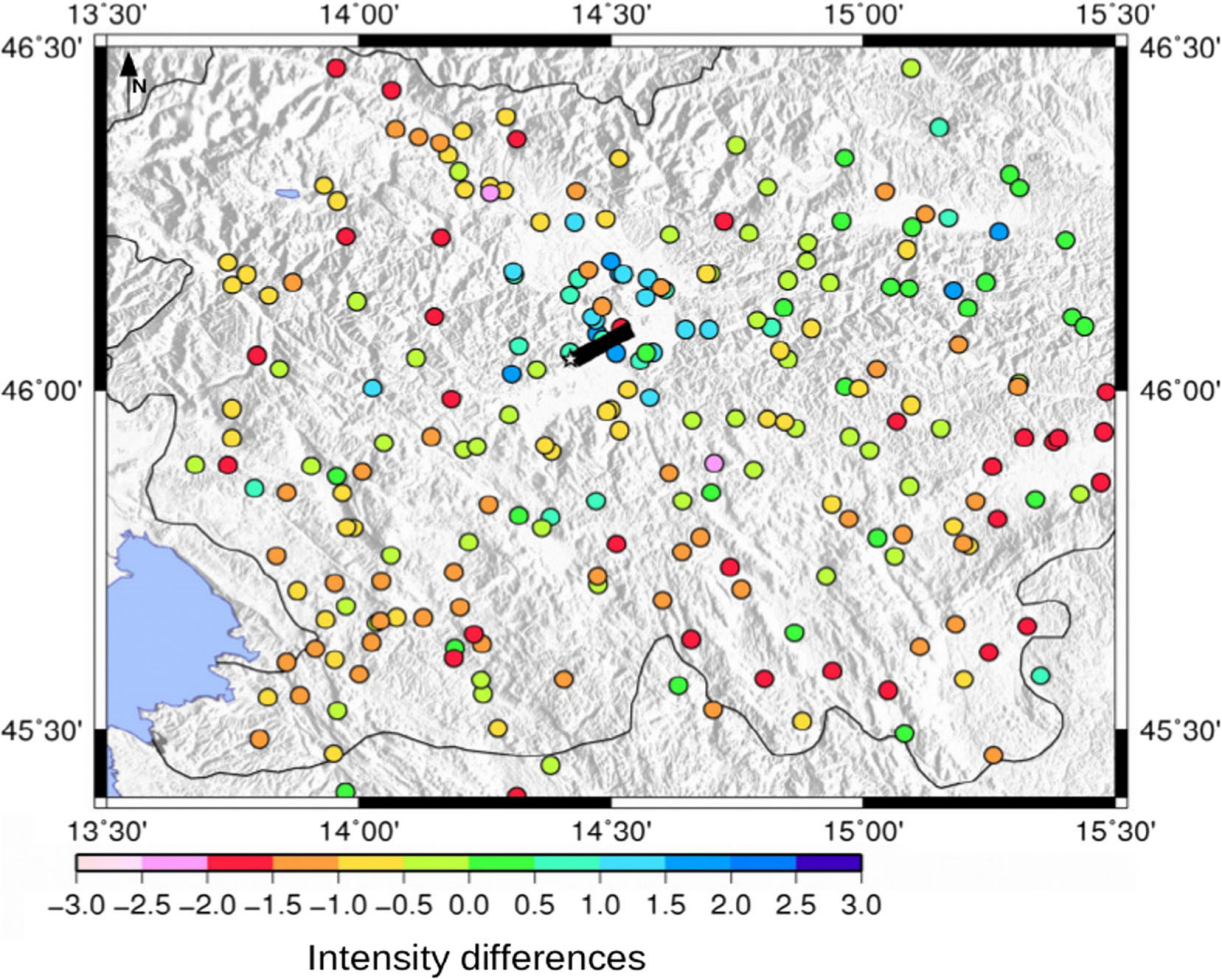
Map reporting the distribution of the intensity differences (*I*_observed_ − *I*_calculated_) for the scenario using as generating fault the Vic one

The calculated intensity data points obtained by using as causative fault the Ortnek fault, with a rupture propagating toward NW with respect to the fault and a moment magnitude of 5.4, have the minimum of both intensity differences and misfit values, as presented in Fig. 6. Another candidate for the causative fault could also be the nearby Želimlje fault (Table 4). Therefore, from these ground motion scenario computations and analyses, considering as input data only the intensity data points, a strike-slip fault with a moment magnitude of 5.4 located to the south of the city could be considered as the most probable generating fault and moment magnitude value for the 1895 Ljubljana event. It is interesting to analyze in detail the map of the differences (*I*_observed_ − *I*_calculated_) for this selected scenario (Fig. 7) comparing it with the differences map using as causative fault, the Vic one, a reverse fault at the north of Ljubljana (Fig. 8). The intensity differences in Fig. 8 are higher than those in Fig. 7, where basically most of the differences are almost close to 0, except for the part north of Ljubljana. This could be due to the fact that in this study the site effects are not included in the simulations. In fact, this zone with positive differences, indicating an underestimation of the PGV1Hz values, is characterized by the Quaternary sediments of the Ljubljana Basin reaching thicknesses of up to 270 m (Žlebnik 1971). Intensity point site classification made by Sirovich et al. (2012) show the area is characterized mainly by C1 (soft sediments with thickness over 30 m), mixed soil type, and C2 (soft sediments with thickness of 5–30 m) (Fig. 3), which contributed to amplification of earthquake ground motion.

## Conclusions

For each of the candidate faults (Vič, Želimlje, Borovnica, Vodice, Ortnek, Mišjedolski, Dobrepolje), PGV is estimated using four different ground motion prediction equations (Massa et al. 2008; Akkar and Bommer 2010; Bindi et al. 2014; Cauzzi et al. 2014). In addition, several ground motion scenarios are produced in terms of PGV1Hz, varying the rupturing direction and the seismic moment value. The calculated intensity data points for each set of PGV and PGV1Hz values are obtained applying GMICEs, expressly calculated for this study.

The quantitative comparison is made between the observed IDPs from the ARSO macroseismic archive and the calculated IDPs using GMPEs and ground motion scenarios. For the GMPE results, it is not possible to distinguish which one could be the causative fault of the Ljubljana event. The different PGV sets are comparable for each studied fault in terms of average intensity differences and misfits. This could be due to the fact that the GMPE fault term seems not to have a relevant weight in the PGV value determination, while the most relevant part of the value depends on the distance. The studied area and the chosen faults are close to each other, so probably the GMPE application is not the most adequate for this study. It is also necessary to point out the potential impact of the directivity not included in the GMPEs considered in this study.

However, the intensity data points help to highlight the possible fault mechanism and location of the causative fault. In fact, despite the frequency limitation of 1 Hz, the comparison between observed and calculated intensities, obtained from ground motion scenarios, points toward a strike-slip fault, located to the south of Ljubljana, with a rupture propagating toward the NW and a moment magnitude of 5.4, as the most probable generating fault of the 1895 Ljubljana earthquake.

### Data availability

ARSO 2012 macroseismic archive. Agencija Republike Slovenije za Okolje, Ljubljana, Slovenia, electronic database

The Italian Macroseismic Database DBMI11 (Locati et al. 2011) for the seismic events until 2002: http://emidius.mi.ingv.it/DBMI11/ (last accessed July 2016)

The Italian Macroseismic Database DBMI15 (Locati et al. 2016) for the seismic events from 2003 until 2014: http://emidius.mi.ingv.it/DBMI15/ (last accessed March 2017)

## Electronic supplementary material

ESM 1 (DOC 450 kb)
