# Supplementary material for: The 1895 Ljubljana earthquake: can the intensity data points discriminate which one of the nearby faults was the causative one?
Source: J Seismol. 2018 May 30;22(4):927–41. doi: 10.1007/s10950-018-9743-z (PMC6182731; doi:10.1007/s10950-018-9743-z)

**The 1895 Ljubljana earthquake: can the intensity data points discriminate which one of the nearby faults was the causative one?**

Lara Tiberi, Giovanni Costa, Petra Jamšek Rupnik, Ina Cecić and Peter Suhadolc

**Table S1.** Summary of the source mechanisms of the eight possible causative faults of the 1895 Ljubljana event.

| **Faults** | **Strike (°)** | **Dip (°)** | **Rake (°)** | **References** |
| --- | --- | --- | --- | --- |
| Vič | 235-250 | 45-80 | 60-90 | Grad and Ferjančič (1974), Premru (1982),Verbič (2006) |
| Želimlje | 320-345 | 70-85 | 160-180 | Buser (1969,1974) |
| Borovnica | 300-330 | 70-85 | 160-180 | Buser et al. (1967), Buser (1970) |
| Jukić (2009) | 282 | 38 | 86 | Jukić (2009) |
| Vodice (N - segment) | 275 | 55 | 90 | Jamšek Rupnik et al.(2013) |
| Vodice (S - segment) | 255 | 55 | 90 | Jamšek Rupnik et al.(2013) |
| Ortnek | 320-340 | 70-85 | 160-180 | Buser (1969,1974) |
| Mišjedolski | 320-340 | 70-85 | 160-180 | Buser (1969, 1974) |
| Dobrepolje | 290-340 | 70-85 | 160-180 | Buser (1969, 1974) |

**Table S2.** Data set used for the regression laws estimation. The data are taken from the CE3RN - Central Eastern European Earthquake and Research Network (Costa et al., 2010, Bragato et al., 2014) and the Italian RAN - Rete Accelerometrica Nazionale databases (Gorini et al., 2010; Costa et al., 2015).

| **Time (mm/dd/yyyy hh:mm)** | **Latitude (°)** | **Longitude (°)** | **Depth (km)** | **Ml** |
| --- | --- | --- | --- | --- |
| 02/14/2002 3:18 | 46.44 | 13.12 | 15 | 4.5 |
| 07/12/2004 13:04 | 46.31 | 13.62 | 11 | 4.9 |
| 01/14/2005 7:58 | 46.2 | 14.03 | 19 | 4.1 |
| 04/24/2005 18:34 | 45.56 | 14.27 | 16 | 3.8 |
| 02/05/2007 8:30 | 45.09 | 14.99 | 19 | 4.1 |
| 12/23/2008 15:24 | 44.50 | 10.38 | 31 | 5.1 |
| 04/06/2009 01:32 | 42.35 | 13.38 | 10 | 5.8 |
| 12/15/2009 13:11 | 43.007 | 12.271 | 9 | 4.2 |
| 12/19/2009 9:01 | 37.807 | 14.865 | 32 | 4.4 |
| 08/16/2010 12:54 | 38.376 | 14.974 | 14 | 4.7 |
| 06/23/2011 22:02 | 38.064 | 14.784 | 7 | 4.7 |
| 07/17/2011 18:30 | 45.01 | 11.387 | 2 | 4.8 |
| 07/25/2011 12:31 | 45.016 | 7.365 | 11 | 4.7 |
| 01/25/2012 08:06 | 44.871 | 10.51 | 29 | 4.9 |
| 12/03/2012 04:36 | 46.23 | 14.78 | 14 | 3.8 |
| 10/25/2012 23:05 | 39.874 | 16.015 | 10 | 5.3 |
| 01/04/2013 7:50 | 37.879 | 14.727 | 16 | 4.4 |
| 06/21/2013 10:33 | 44.142 | 10.111 | 10 | 5.4 |
| 08/15/2013 23:06 | 38.182 | 14.829 | 17 | 4.7 |
| 12/29/2013 17:08 | 41.388 | 14.451 | 15 | 5.1 |
| 04/22/2014 8:58 | 45.65 | 14.25 | 23 | 3.9 |
| 08/29/2015 18:47 | 46.32 | 13.61 | 5 | 3.9 |
| 11/01/2015 7:52 | 45.85 | 15.54 | 5 | 4.2 |
| 02/08/2016 17:08 | 36.980 | 14.858 | 9 | 4.6 |

**Table S3. Summary of the conversion between the soil classification classes presented in the Sirovich et al. (2012) work and the soil characteristics coefficient values for each GMPE used.**

| Sirovich et al. (2012) | Massa et al. (2008) | Akkar et al. (2010) | Bindi et al. (2014)  Cauzzi et al. (2014) |
| --- | --- | --- | --- |
| A | Sa = 1; S(B+c) = 0 | Ss = 0; Sa = 1 | Sb = Sc = Sd = 0 |
| B | Sa = 1; S(B+c) = 0 | Ss = 0; Sa = 1 | Sb = 1; Sc = Sd = 0 |
| C1 | Sa = 0; S(B+c) = 1 | Ss = 1; Sa = 0 | Sb = Sd = 0; Sc = 1 |
| C2 | Sa = 0; S(B+c) = 1 | Ss = 1; Sa = 0 | Sb = Sc = 0, Sd = 1 |

**Table S4.** Summary of the source parameters of the eight possible causative faults of the 1895 Ljubljana event used in the ground motion scenarios.

| **Fault** | **Depth (km)** | **L (km)** | **W (km)** | **Strike (°)** | **Dip (°)** | **Rake (°)** | **NP (lat, lon)** |
| --- | --- | --- | --- | --- | --- | --- | --- |
| Vič 1 | 6 | 10 | 5 | 240 | 60 | 80 | (46.01, 14.34) |
| Vič 2 | 6 | 10 | 5 | 240 | 60 | 80 | (46.06, 14.45) |
| Vič 3 | 6 | 10 | 5 | 240 | 60 | 80 | (46.05, 14.42) |
| Vič 4 | 6 | 10 | 5 | 240 | 60 | 80 | (46.09, 14.53) |
| Želimlje 1 | 7 | 10 | 5 | 335 | 80 | 170 | (46.02, 14.52) |
| Želimlje 2 | 7 | 10 | 5 | 335 | 80 | 170 | (45.93, 14.57) |
| Borovnica 1 | 7 | 10 | 5 | 315 | 80 | 170 | (45.99, 14.34) |
| Borovnica 2 | 7 | 10 | 5 | 315 | 80 | 170 | (45.91, 14.39) |
| Jukić(2009) | 6 | 15 | 6 | 282 | 38 | 86 | (46.10, 14.28) |
| Vodice N | 6 | 10 | 5 | 275 | 55 | 90 | (46.23, 14.40) |
| Vodice S | 6 | 11 | 5 | 255 | 55 | 90 | (46.20, 14.39) |
| Ortnek | 7 | 10 | 5 | 335 | 80 | 170 | (45.95, 14.57) |
| Mišjedolski | 7 | 10 | 5 | 335 | 80 | 170 | (45.94, 14.51) |
| Dobropolje | 7 | 10 | 5 | 315 | 80 | 170 | (45.88, 14.69) |

From the left to the right: the studied fault, the hypothetical depth of the event, the length of the fault (L), the fault width (W), strike, dip, rake and the nucleation point location (NP).

**Figure S1.** “Synthetic” regression laws with the synthetic PGV1Hz values VS the “real” regression

law. The image: (a) is the simulation using the Vič fault type 1, 2 (b), 3 (c) and 4 (d) respectively; (e) and (f) using the Želimlje fault type 1 and 2 respectively; (g) is for the Borovnica

fault type 1 and 2 (h); (i) is the Jukić (2009) inversion resulting fault; the (l) and (m) are the north and the south segment of the Vodice fault; the (n) is the result using as causative the Ortnek fault; (o) the Mišjedolski and (p) the Dobrepolje fault.


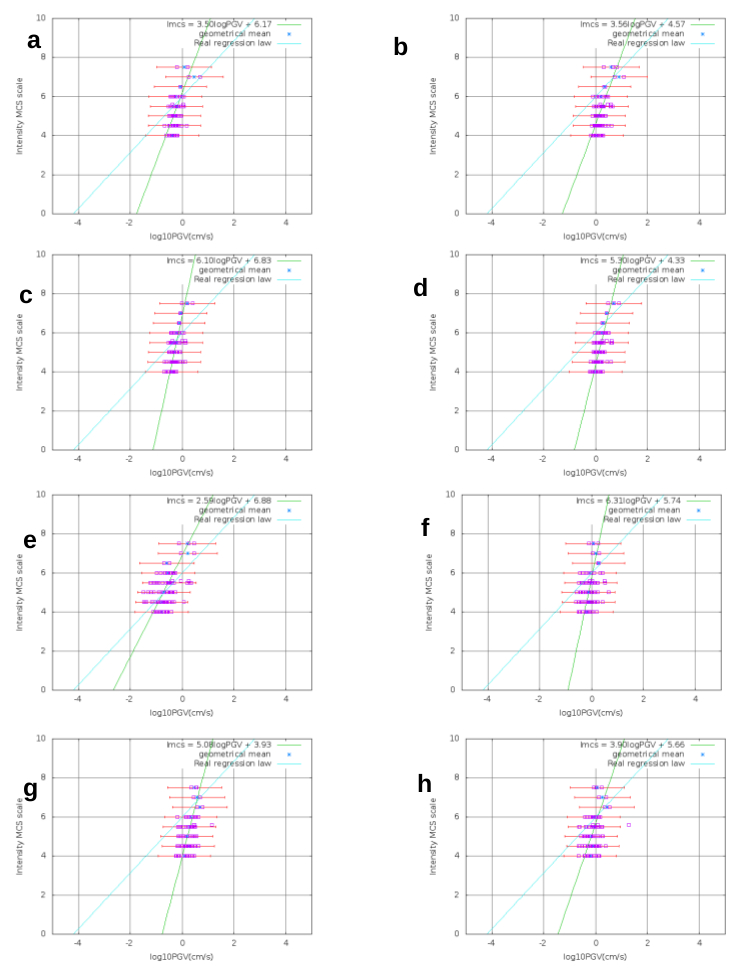


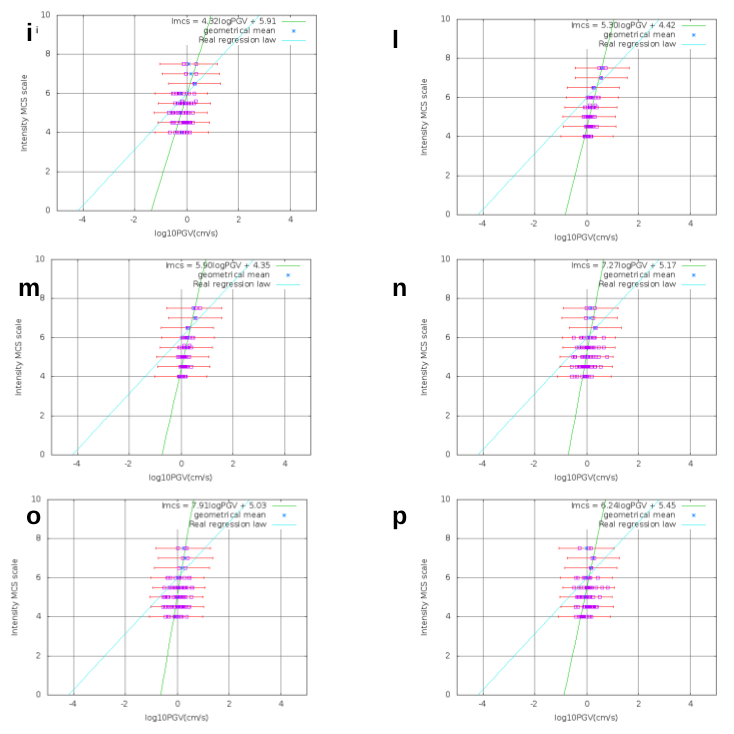

Supplement: Supplementary file 1 — (DOC 450 kb) [file 10950_2018_9743_MOESM1_ESM.doc]
